# Brain Fatty Acid Binding Protein (Fabp7) Is Diurnally Regulated in Astrocytes and Hippocampal Granule Cell Precursors in Adult Rodent Brain

**DOI:** 10.1371/journal.pone.0001631

**Published:** 2008-02-20

**Authors:** Jason R. Gerstner, Quentin Z. Bremer, William M. Vander Heyden, Timothy M. LaVaute, Jerry C. Yin, Charles F. Landry

**Affiliations:** 1 Neuroscience Training Program, University of Wisconsin-Madison, Madison, Wisconsin, United States of America; 2 Department of Genetics, University of Wisconsin-Madison, Madison, Wisconsin, United States of America; 3 Department of Psychiatry, University of Wisconsin-Madison, Madison, Wisconsin, United States of America; University of Washington, United States of America

## Abstract

Brain fatty acid binding protein (Fabp7), which is important in early nervous system development, is expressed in astrocytes and neuronal cell precursors in mature brain. We report here that levels of Fabp7 mRNA in adult murine brain change over a 24 hour period. Unlike Fabp5, a fatty acid binding protein that is expressed widely in various cell types within brain, RNA analysis revealed that Fabp7 mRNA levels were elevated during the light period and lower during dark in brain regions involved in sleep and activity mechanisms. This pattern of Fabp7 mRNA expression was confirmed using *in situ* hybridization and found to occur throughout the entire brain. Changes in the intracellular distribution of Fabp7 mRNA were also evident over a 24 hour period. Diurnal changes in Fabp7, however, were not found in postnatal day 6 brain, when astrocytes are not yet mature. In contrast, granule cell precursors of the subgranular zone of adult hippocampus did undergo diurnal changes in Fabp7 expression. These changes paralleled oscillations in Fabp7 mRNA throughout the brain suggesting that cell-coordinated signals likely control brain-wide Fabp7 mRNA expression. Immunoblots revealed that Fabp7 protein levels also underwent diurnal changes in abundance, with peak levels occurring in the dark period. Of clock or clock-regulated genes, the synchronized, global cycling pattern of Fabp7 expression is unique and implicates glial cells in the response or modulation of activity and/or circadian rhythms.

## Introduction

Brain fatty acid binding protein (also known as brain lipid binding protein or Fabp7) is a member of a large family of fatty acid binding proteins (Fabps) of relatively small molecular weight (∼15 kD) that are expressed in a diverse set of vertebrate and invertebrate tissues [1]. Fabps, which facilitate the solubility of hydrophobic long chain fatty acids and function primarily in fatty acid uptake and transport [2], have been widely implicated in cell growth and differentiation [3]–[7]. In the central nervous system (CNS), three Fabp proteins with different cell-type distributions have been identified: Heart-type Fabp (Fabp3), epidermal-type Fabp (Fabp5), and brain-type Fabp, Fabp7 [8], [9]. Fabps within brain are thought to govern the uptake and delivery of fatty acids like docosahexanoic acid (DHA) and arachadonic acid and to play important roles in cell differentiation [3], [10], [11].

The initial identification of Fabp7 established its presence within radial glia in embryonic brain and in neuronal cell progenitors in mature brain [3], [12], [13]. In fact, most neuronal cell populations are thought to be derived from Fabp7-expressing progenitors [14]. The regulation of Fabp7 mRNA expression has been shown to be downstream of Notch signaling [15], and dependent on Pax6 [14], [16] and POU/Pbx transcription factors [17]. Fabp7 mRNA levels were found to peak at birth and to undergo a dramatic reduction during early postnatal development, but to persist in radial glia-like neuronal progenitors and specific mature astrocyte populations [13]. Although the role of Fabp7 in fatty acid delivery during development is not clear, its involvement in cell growth and differentiation has been implicated based on its influence on cell morphology [3]. Further, an induction of Fabp7 increases the motility of glioma cells [18].

Recently it has been shown that the targeted deletion of Fabp7 results in an enhancement of fear memory and anxiety in adulthood [9]. Interestingly, the ability of the fatty acid, DHA to modulate NMDA receptor activation in hippocampal neurons is eliminated in these Fabp7-mutant mice. Whether these effects are due to cell-intrinsic changes that occur in the absence of Fabp7 during neuronal cell precursor maturation or are a result of an absence of Fabp7 in mature astrocytes is not known.

Changes in the expression of molecules involved in the regulation of the circadian cycle have been shown to have strong functional and behavioral consequences. For example, mutations in various clock genes have been shown to alter feeding behavior [19]–[22], long-term memory formation [23], [24] and to exert strong effects on drug conditioning behavior in both flies and mice [25]–[27]. We have previously shown that Fabp7 also undergoes diurnal changes in expression in hypothalamus [28]. Given the potential role of Fabp7 in neurogenesis and anxiety mechanisms, and the role of circadian genes in complex behavior, we examined the expression of Fabp7 throughout the brain and found that it underwent global and coordinated diurnal regulation in astrocytes. Further, this synchronized cycling pattern in astrocytes was also observed in granule cell progenitors of the hippocampus.

## Results

### Fabp7 mRNA is diurnally expressed in brain regions involved in the regulation of arousal

**Figure 1:**
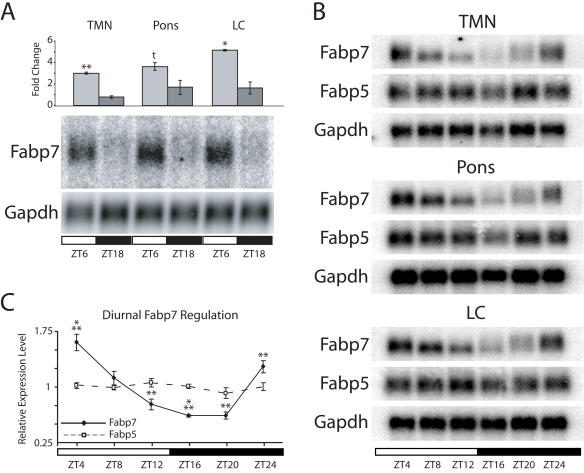
Fabp7 mRNA is diurnally regulated in brain regions involved in sleep and activity. A) RNA collected from tuberomammillary nucleus (TMN), pons and locus coeruleus (LC) subjected to Northern blotting revealed elevated levels of Fabp7 mRNA at Zeitgeber time (ZT) 6 compared to ZT18 (bottom panel). Glyeraldehyde-3-phosphate dehydrogenase (Gapdh) was used as a loading control. Graphic representation of Fabp7 expression normalized against Gapdh indicated 3 to 5 fold changes in mRNA levels (top panel); **p<0.01, *p<0.05, t = 0.06 (t-test). Each time point represents 12 individual animals pooled. *N* = 2–3 groups. B) Northern blot analysis of RNA collected every 4 hours showed diurnal changes in Fabp7 mRNA but not Fabp5. C) Collapsed data from B normalized with Gapdh indicated significant diurnal changes in Fabp7 mRNA across a 24-hour period but no changes in Fabp5 expression. One-way ANOVA, ***p<0.001, **p<0.01 (post-hoc Bonferroni). Each value represents the regional average +/− S.E.M. Each time point represents 3 individual animals pooled.

Fabp7, a protein present in astrocytes and related cell types [3], [12], [13], undergoes diurnal changes in mRNA abundance in regions of murine hypothalamus that are involved in sleep/wake regulation and circadian rhythmicity [28]. Fabp7 mRNA expression also cycles over a 48 hour period within the suprachiasmatic nucleus, where oscillations persisted in dark (free-running) conditions [29], [30]. To determine whether Fabp7 mRNA is diurnally expressed in other brain regions involved in activity, we examined the cycling of Fabp7 mRNA in the tuberomammilary nucleus (TMN), which contains histaminergic neurons thought to play a primary role in brain arousal [31]–[33], the pons, a brain area that has a broad role in activity states and the locus coeruleus (LC), which contains norepinephrine-releasing cells important in regulating sleep and activity [34]. Within these three regions, Fabp7 mRNA levels at Zeitgeber time (ZT) 6 (the middle of the light period) were three to five-fold higher than ZT18 (the middle of the dark period; Fig. 1A). Detailed time point analysis of TMN, pons and LC further revealed a gradual decrease in Fabp7 mRNA during the light period followed by an increase in levels at the end of dark (Fig. 1B). The diurnal pattern of Fabp7 mRNA expression was strikingly similar in all three brain regions and was identical to the diurnal change in Fabp7 mRNA levels evident in hypothalamus [28].

The diurnal pattern of Fabp7 mRNA expression was compared to epidermal-type fatty acid binding protein, Fabp5, which is expressed in a number of cell types in brain including astrocytes [10], [11]. Unlike Fabp7, Fabp5 mRNA was not found to cycle in the TMN, pons or LC (Fig. 1B). Collapsed data from figure 1B is depicted as relative signal intensity in figure 1C to illustrate differences in diurnal expression between Fabp7 and 5. Significant differences were evident in Fabp7, but not Fabp5, mRNA levels across the diurnal cycle. The other Fabp expressed in brain, heart-type fatty acid binding protein (Fabp3), which is exclusive to neurons, was also not found to cycle (data not shown). The pattern of Fabp7 expression in diurnal samples of RNA from pons subjected to quantitative RT-PCR (qRT-PCR) closely matched Northern blotting data (Fig. S1).

To determine whether pre-processed heteronuclear (hn) Fabp7 RNA underwent similar changes in abundance, we used qRT-PCR to examine the relative concentrations of Fabp7 intron 1 in TMN, pons and LC across a diurnal profile (Fig. S1). These results indicated that higher intron 1 hnRNA levels were generally present around the dark-to-light period transition. *In situ* hybridization with probe to intron 1 confirmed qRT-PCR results (data not shown). These results suggest that the increase in Fabp7 mRNA that occurs near the end of the dark period is due to enhanced transcriptional activity.

### Diurnal changes in Fabp7 mRNA occur throughout the brain

**Figure 2:**
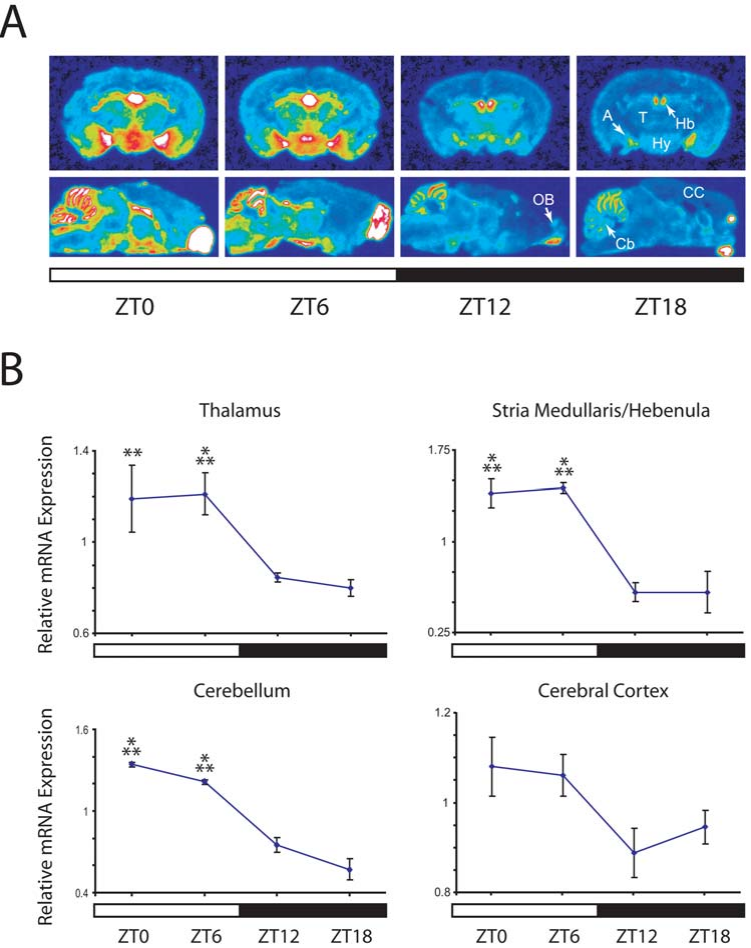
Fabp7 mRNA changes globally during the diurnal period. A) Coronal (top series) and sagittal (bottom series) sections from four diurnal time points subjected to *in situ* hybridization using ^35^S-labeled Fabp7 antisense riboprobe. Note more intense expression levels during lights-on. B) Graphic representation of relative Fabp7 mRNA expression at four time points. A statistically significant diurnal variation in expression was observed; Two-way ANOVA (p<0.001). ***p<0.001, **p<0.01 versus ZT18 (post-hoc Bonferroni). Each value represents the average +/− S.E.M. *N* = 2–5 per time point. A, Amygdala; Cb, cerebellum; CC, cerebral cortex; Hb, medullaris/hebenula; Hy, hypothalamus; OB, olfactory bulb; T, thalamus

*In situ* hybridization was used to examine the diurnal profile of Fabp7 mRNA expression in more detail. Highest signal intensities were evident during the light period in agreement with results using Northern blotting and qRT-PCR RNA analyses (Fig. 2A). At all time points, signal within coronal sections at the level of dorsal hippocampus showed highest intensities in stria medullaris/hebenula (Hb), amygdala (A) and hypothalamus (Hy). Sagittal sections confirmed the diurnal cycling of Fabp7 mRNA and indicated high levels in cerebellum (Cb) and olfactory bulb (OB). Densitometric measurements from a number of brain regions that included thalamus (T), Hb, cerebral cortex (CC) and Cb revealed the diurnal regulation of Fabp7 expression to include a broader pattern that extended beyond regions known to specifically influence sleep/wake or circadian rhythmicity.

Given that the recently described Chordc1 mRNA has been shown to cycle globally in brain during early postnatal development [35], and that Fabp7 mRNA is abundant in rat [12] and mouse brain (Fig. S2A), we examined Fabp7 mRNA expression at postnatal day 6, when many astrocytes have completed migration and are beginning to mature [36]. Unlike adult brain, Fabp7 mRNA was not found to cycle in P6 brain (Fig. S2B).

### Fabp7 protein levels are elevated during the dark period

**Figure 3:**
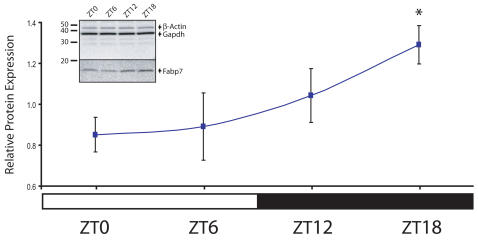
Fabp7 protein levels are elevated during the dark period. Graphic representation of Western blots from whole brain indicating an increase in Fabp7 protein levels with increasing Zeitgeber time. There is a significant increase in Fabp7 protein at ZT18 compared to ZT0,*p<0.05 (*t*-test). Each value represents the average +/− S.E.M. *N* = 3–4 per time point. Values are normalized to an average of Gapdh and β-actin. Inset: Representative immunoblot of whole brain homogenates from tissue isolated at four diurnal time points. A single, predicted 15 kilodalton band was evident in blots incubated with anti-Fabp7 antibody. Blots were subsequently incubated with anti-β-actin and anti-glyceraldehyde-3-phosphate dehydrogenase (Gapdh).

Immunoblotting was used to determine whether changes in Fabp7 mRNA levels resulted in changes in Fabp7 protein. Since Northern blotting and *in situ* hybridization indicated that Fabp7 mRNA levels underwent the same pattern of diurnal regulation regardless of brain region, we examined Fabp7 protein levels in whole brain extracts collected at four diurnal time points. Like Fabp7 mRNA, Fabp7 protein levels were found to differ depending on time of day (Fig. 3). The highest amounts of protein were evident at ZT18 and were statistically different than the lowest amounts found at ZT0 (t-test, p<0.05). Higher Fabp7 protein levels during the dark period were in contrast with mRNA levels, which were highest during the day suggesting that Fabp7 protein accumulation lagged peak Fabp7 mRNA levels by about 12 hours.

### Fabp7 mRNA is diurnally regulated in amygdala and may undergo changes in intracellular distribution during the light-dark cycle

**Figure 4:**
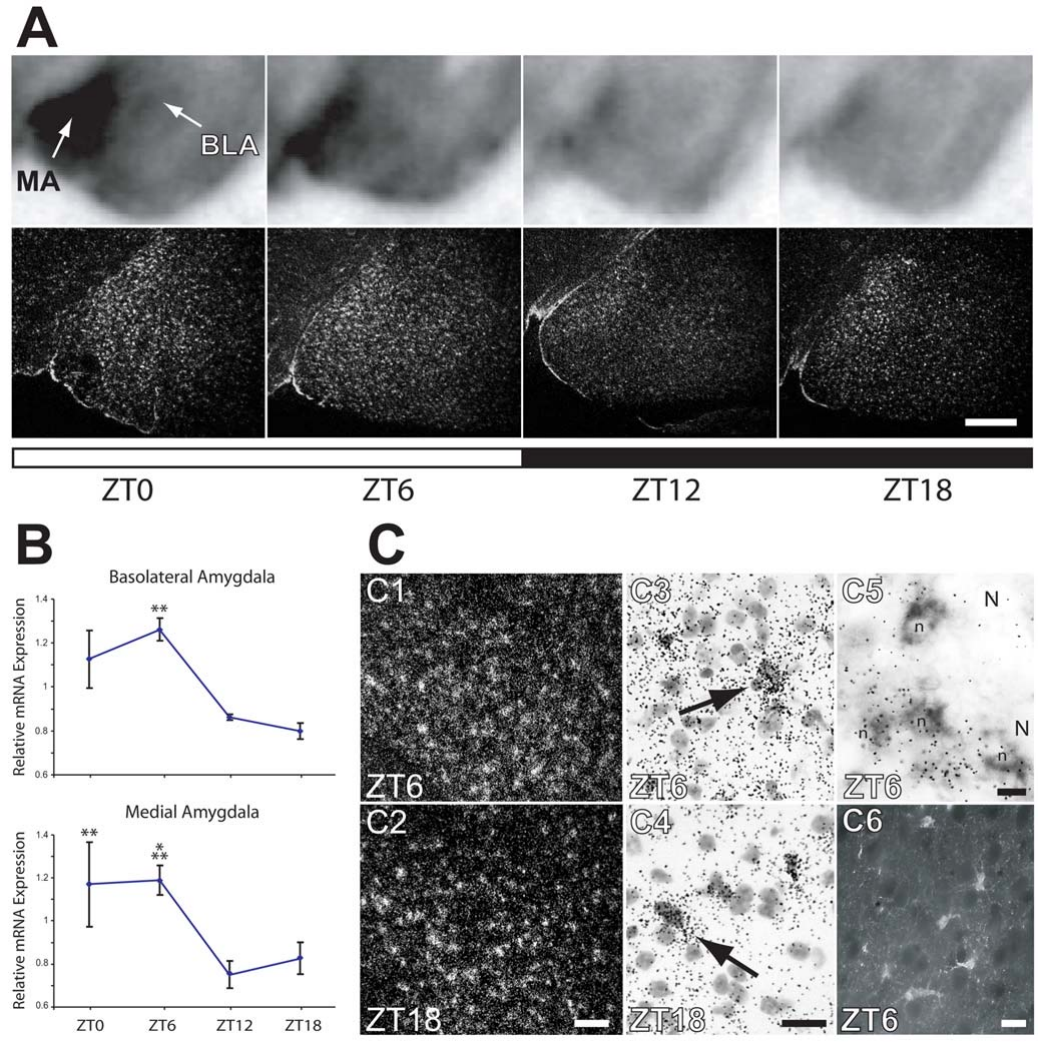
The amount and distribution of Fabp7 mRNA in amygdala changes during the diurnal cycle. A) *In situ* hybridization of coronal tissue sections probed for Fabp7 mRNA at four diurnal time points. Top panels, phosphorimages of amygdala, bottom panels, dark field images of a different set of hybridized sections subjected to emulsion autoradiography. Higher levels of Fabp7 were evident during lights-on. B) Graphic representation of elevated Fabp7 mRNA levels during lights-on in basolateral and medial amygdala. Two-way ANOVA (p = 0.002). ***p<0.001, **p<0.01 vs. ZT18 (post-hoc t-test). Each value represents the average +/− S.E.M. *N* = 2–4 per time point. C) Sections processed for *in situ* hybridization for Fabp7 subjected to emulsion autoradiography. Dark field microscopy revealed a more dispersed labeling pattern at ZT 6 (C1) compared to ZT18 (C2). Higher magnification micrographs of Nissl counterstained sections indicated labeling over cells whose staining pattern was consistent with an astrocyte morphology and whose labeling pattern was more disperse at ZT6 (C3) compared to ZT18 (C4). Note that silver grain accumulation around cell nuclei was similar at the two time points (arrows in C3 and C4). Astrocytes stained with DIG-labeled riboprobe for Fabp7 (unstained astrocytic nuclei (n) are indicated) were also co-labeled for mRNA for the astrocyte protein, GFAP (silver grain accumulations). Unstained neuronal nuclei (N) are shown for reference. A representative tissue section subjected to immunohistochemistry with anti-Fabp7 antibodies is shown in C6. Note astrocytic processes emanating from stained cell somata. Bar in A = 400 µm; in C1,C2 = 100 µm; in C3,C4 = 20 µm; in C5 = 10 µm, C6 = 30 µm BLA, basolateral amygdala; MA, medial amygdala

Fabp7 has recently been implicated in fear memory and anxiety [9] and we therefore examined the region-specific distribution of Fabp7 mRNA in amygdala and hippocampus. In amygdala, Fabp7 mRNA cycled identically to other brain regions with highest mRNA levels present in the light period (Fig. 4). This distinctive pattern of expression was evident in both basolateral (BLA) and medial (MA) nuclei (Fig. 4A) where significant changes in mRNA levels oscillated throughout the diurnal cycle (Fig. 4B). More detailed analysis using emulsion autoradiography confirmed elevated levels of Fabp7 mRNA during lights-on (compare Fig. 4C1 and C2). Higher magnification analysis of BLA revealed that silver grain distribution corresponding to Fabp7 mRNA was more disperse at ZT6 compared to ZT18 (compare 4C3 and C4). Note the more dense accumulation of silver grains around cell somata at ZT18 (arrow in 4C4) compared to ZT6 (arrow in 4C3). A more dispersed distribution of sliver grains was common throughout the brain toward the end of the light period (data not shown) and these observations suggest that the diurnal change in Fabp7 mRNA abundance may coincide with changes in mRNA targeting. The pattern of expression of Fabp7 mRNA was consistent with an astrocytic labeling pattern as has been shown previously [9] and cells that stained with DIG-labeled Fabp7 riboprobe were also found to co-label with radiolabeled probe to the astrocyte mRNA, Glial fibrillary acidic protein (4C5). Immunostained images of Fabp7-expressing astrocytes in BLA are shown for reference (Fig. 4C6).

### Diurnal changes in Fabp7 mRNA levels in hippocampus are also present in granule cell precursors

**Figure 5:**
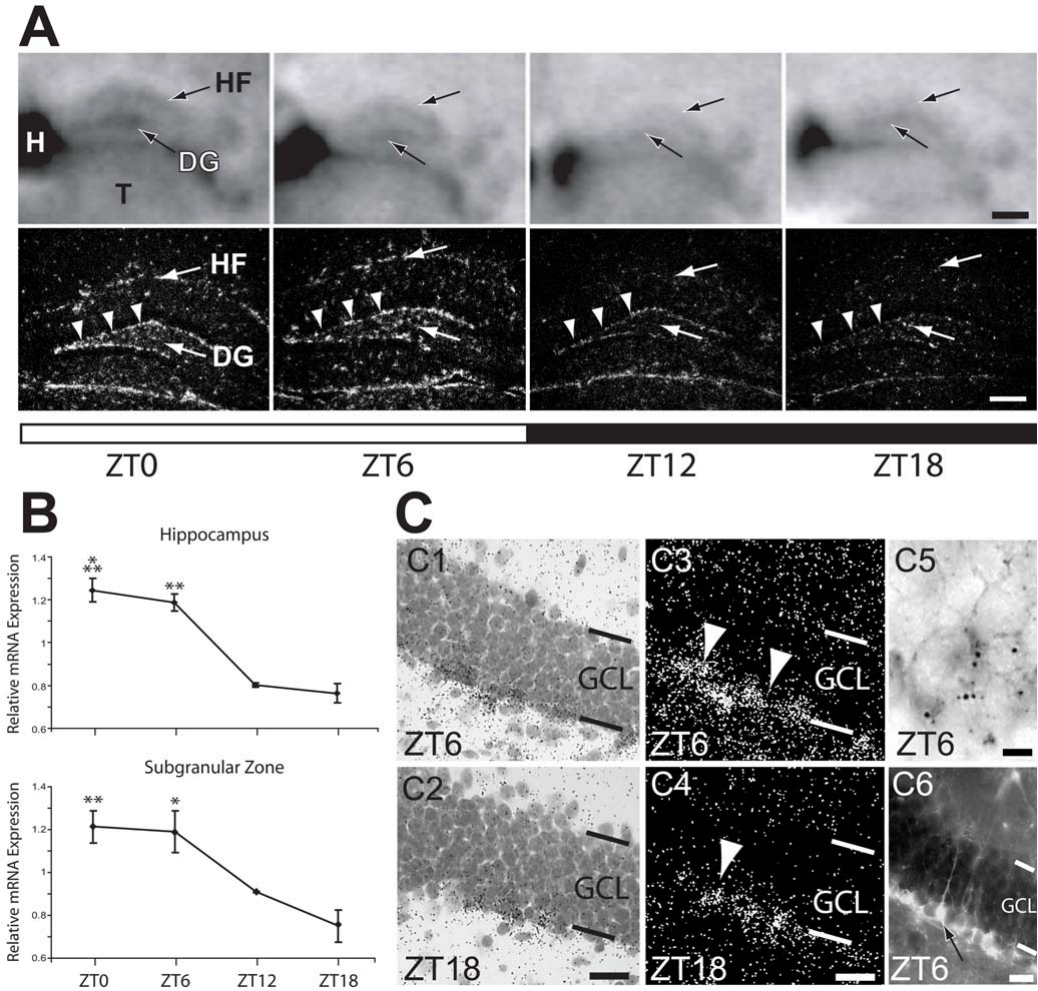
Fabp7 mRNA expression is diurnally regulated in hippocampus and subgranular zone of dentate gyrus. A) *In situ* hybridization of coronal tissue sections probed for Fabp7 mRNA at four diurnal time points. Top panels, phosphorimages of hippocampus, bottom panels, dark field images of a different set of hybridized sections subjected to emulsion autoradiography. Higher levels of Fabp7 were evident during lights-on. Arrowheads in bottom panels indicate subgranular zone of dentate gyrus (DG). B) Graphic representation of *in situ* hybridization data indicating elevated levels of Fabp7 mRNA during lights-on in hippocampus (top panel) and subgranular zone (bottom panel). One-way ANOVA (p<0.001). ***p<0.001, **p<0.01 vs. ZT18 (post-hoc Bonferroni). Each value represents the average +/− S.E.M. *N* = 2–4 per time point. C) Sections subjected to *in situ* hybridization for Fabp7 and emulsion autoradiography. Light- (C1, C2) and dark field (C3, C4) micrographs of sections from ZT6 and ZT18, respectively. Arrowheads indicate Fabp7 positive precursor cells in subgranular zone. Sections co-stained for Fabp7 mRNA (dark stain) and nestin mRNA (dark silver grains) identified co-labeled precursors in the subgranular zone (C5). Sections subjected to immunohistochemistry for Fabp7 (C6) revealed radial fibers emanating from stained cell bodies (arrows) as previously described. Bar in upper, lightfield micrographs in A = 500 µm; in lower, darkfield micrographs in A = 200 µm; in C1,C2 = 30 µm; in C3,C4 = 30 µm; in C5 = 5 µm; C6 = 25 µm. DG, dentate gyrus; GCL, granule cell layer; H, hebenula; HF, hippocampal fissure; T, thalamus

Fabp7 mRNA was also diurnally regulated in hippocampus with higher relative levels of expression present in the hippocampal fissure (HF) and dentate gyrus (DG; Fig. 5A). Expression of Fabp7 was pronounced in the subgranular zone (SGZ) of DG and was higher during lights-on (arrowheads in Fig. 5A). These changes were found to be significant in the hippocampus as well as within the granule cell precursor layer (Fig. 5B).

Higher resolution autoradiograpy confirmed intense expression of Fabp7 mRNA in cells within the SGZ that was absent from the granule cell layer (GCL; Fig. 5C). Silver grain accumulation over cells within this region (arrowheads in Fig. 5C3 and C4) indicated higher Fabp7 expression at ZT6. Granule cell precursors within this layer, which are known to express Fabp7 [37], give rise to granule neurons throughout the life of the animal [38]. These cells were found to co-express Fabp7 mRNA (note Fabp7-stained precursor in Fig. 5C5) and mRNA for the intermediate filament protein, Nestin as previously described [37]. Precursor cells within the SGZ were also strongly Fabp7 immunoreactive (Fig. 5C6). Since Fabp7 is confined to radial glia-like type-1 and proliferative type-2 cells within the SGZ [38], our results indicate that Fabp7 mRNA levels oscillate within these cells. Further, we identified lower levels of Fabp7 expression within the granule cell layer or directly below the SGZ in agreement with previous observations [37] suggesting that the presence of Fabp7 in astrocytes within the granule cell region did not contribute significantly to our analysis of SGZ. Interestingly, we found that cells of the SGZ underwent diurnal changes in Fabp7 mRNA abundance that paralleled the regulation of Fabp7 expression in the hippocampus proper as well as throughout the brain (lower panel in 5B). These results suggest that Fabp7 mRNA undergoes synchronized diurnal changes in expression in multiple cell-types in the brain.

## Discussion

This report describes the diurnal regulation of brain fatty acid binding protein (Fabp7) within astrocytes and subgranular zone precursors of the adult murine brain. Synchronized cycling of Fabp7 mRNA occurred throughout the brain with peak levels present at the beginning of the light period and lowest levels near lights-off. Fabp7 protein was also found to change over a 24 hour period such that an accumulation in protein occurred towards the middle of the dark period when Fabp7 mRNA levels were declining. Interestingly, we observed no changes in Fabp7 mRNA in young postnatal brain but found Fabp7 to cycle in precursor cells of the adult hippocampus. These data suggest that Fabp7 is a novel cycling gene and that its regulation is driven by mechanisms that confer brain-wide, coordinated oscillations in expression.

Given that Fabp7 expression is downstream of Notch signaling [15], Notch-mediated regulation of Fabp7 is a putative mechanism for the oscillation in Fabp7 mRNA that we observe. In fact, a binding site for the Notch effector CBF1 is essential for the transcriptional regulation of Fabp7 in radial glial cells and CBF1 elements within 400 base pairs of the Fabp7 transcription start site are necessary and sufficient for transgene activity in embryonic and postnatal day (P) 6 brain [15]. Further, Notch signaling is thought to synchronize the segmentation clock for somitogenesis where it operates in somite precursor cells of the mesoderm that oscillate through phases of the cell cycle [39]. It is not known whether Notch signaling has a similar role later in development. Also, we did not observe changes in Fabp7 mRNA expression across the light-dark cycle in early postnatal brain (P6) when Notch signaling is known to influence Fabp7 expression [15]. Since astrocyte migration and maturation is not complete at this time in development [36] and because Fabp7 levels are greatly elevated at this age compared to adult [12], it may be that an inability to observe time-of-day changes in Fabp7 mRNA levels is a result of a ceiling affect. Conversely, it may be that the diurnal cycling of Fabp7 mRNA is restricted to mature brain. In addition, other transcriptional regulators, such as Pax6 and POU/Pbx have also been found to influence the developmental profile of Fabp7 expression [16], [17], suggesting alternative regulatory mechanisms for Fabp7 transcription.

Whether Fapb7 expression is tied to known clock mechanisms or is a component of a novel clock pathway is not known. Bioinformatic analyses of the Fabp7 gene suggest an influence of known clock mechanisms given the presence within the proximal Fabp7 promoter of *cis-*elements such as peroxisome proliferater activated receptor (PPRE) and E-box elements. These *cis*-elements play important roles in known clock-related transcriptional regulation [40]. Functional PPREs are present in circadian genes such as BMAL and Rev-erb-alpha [41], [42] and the PPRE-transactivator, peroxisome proliferater activated receptor-alpha (PPARα), is itself regulated by the circadian modulator, CLOCK [43]. In addition, it has been shown that CLOCK:BMAL1 transactivates PPAR target genes via the PPRE [44]. Since CLOCK is known to control the transcription of PPARs, and PPREs are present within the Fabp7 promoter, it may be that the diurnal oscillation in Fabp7 observed in this study is driven by a CLOCK, PPAR-mediated pathway. Further, since other fatty acid binding proteins have been shown to translocate to the nucleus and directly influence PPAR-mediated transcription [45]–[47], and we observe peak Fabp7 protein levels that proceed Fabp7 transcript levels, it is intriguing to speculate that Fabp7 protein influences its own transcription via PPAR. The importance of E-box elements in the diurnal regulation of Fabp7, however, is uncertain since per2, but not Fabp7, has been found to cycle in astrocyte culture [48]; Gerstner and Landry, unpublished observations). Further, unlike Fabp7, circadian genes like CLOCK, per1, per2 and d-albumin binding protein cycle in different temporal phases in different brain regions [49], [50]. It is likely that the regulation of Fabp7 expression results from a combination of PPAR and E-box elements as well as other transcriptional mechanisms. Further study will be required to determine the elements that influence Fabp7 expression in mature brain.

Peak Fabp7 mRNA levels preceded peak protein levels by about 12 hours and a gradual decline in mRNA occurred as protein levels increased. These changes are similar to known circadian and diurnally regulated genes, although the delay that we see here is somewhat longer [51]–[53]. It may be that mechanisms that influence Fabp7 mRNA levels during lights-on are in place to increase the amount of Fabp7 protein during the active (dark) period, perhaps in response to enhanced fatty acid demand at this time. Since high levels in Fabp7 protein in brain during the dark parallel diurnal changes in fatty acid binding proteins in liver and heart, which are also elevated during the active period [54], a mechanism for modulating the delivery or utilization of fatty acids during activity may be a common theme in a number of organ systems. Further, the diurnal rhythmicity of fatty acid binding protein expression in heart and liver are influenced by levels of serum cholesterol [54] implicating fatty acid utilization as a modulating factor in fatty acid binding protein expression.

Diurnal cycling in processes that use fatty acids as functional components may be a common feature of homeostatic mechanisms. For example, fatty acid amide hydrolase (FAAH) activity has been shown to cycle throughout the day [55], and to modulate sleep [56], [57]. Similarly, the fatty-acid metabolites anandamide and 2-arachidonylgycerol have been shown to vary globally across the light-dark cycle [55] and endocannabinoids such as these have been found to participate in the regulation of complex behaviors such as feeding, sleep/wake cycles, and response to stress [58]–[60]. How fatty acids required for these processes are delivered and distributed within cells of the nervous system is not well understood [61] and the cycling of Fabp7 is the first report of an oscillating protein involved in the movement of fatty acids. Diurnal changes in the transport and delivery of fatty acids may therefore be a critical component of lipid-dependant, homeostatic mechanisms.

The brain-wide diurnal regulation of Fabp7 mRNA suggests that either a cell-autonomous mechanism governs its regulation, or that diurnal levels are controlled by a global coordinated signaling system. The diurnal regulation of Fabp7 within granule cell precursors of the hippocampus suggests cell-autonomous mechanisms since these cells are not astrocytes and are only transiently Fabp7 positive [37]. However, since granule-cell precursors are continually born within the subgranular zone [38] where we observed synchronized diurnal expression of Fabp7, we would predict that Fabp7 cycles similarly in precursor cells of different birth dates, indicating a coordinated (non-cell autonomous) system of regulation. Since astrocytes are known to contact subgranular progenitors [62], it is likely that radial glial-like precursor cells are capable of receiving signals from astrocytes. Further, populations of astrocytes have been shown to undergo coordinated activity. For example, astrocytic purinergic signaling has been shown to synchronize pulses of calcium [63], [64] and to modulate heterosynaptic depression through inhibition of presynaptic terminals [65]. Indeed, purinergic signaling by adenosine, as well as lipid-derived prostaglandins, is known to modulate behavioral state, long-term potentiation and long-term depression [66]–[68]. Astrocyte derived calcium signals have also been shown to correlate with neuronal activity [69], and to couple this activity to cerebral microcirculation [70]. That populations of astrocytes are capable of coordinated activity, therefore, further supports the notion that a broad astrocyte-mediated signaling system occurs in brain. Although, the modulation of the cycling of Fabp7 that we observe maybe extrinsic to the brain, i.e., driven by fluctuating serum lipid concentrations that influence astrocyte metabolism, it seems more likely that Fabp7 expression is driven by a yet undefined intrinsic brain signaling system that involves astrocyte communication.

This is the first report to describe a diurnally regulated gene, Fabp7 that cycles globally in astrocytes and related cell-types in the mammalian central nervous system. The global cycling pattern of Fabp7 implicates a coordinated mechanism of regulation that extends to radial glial-like cells of the hippocampal subgranular zone and is consistent with growing evidence that astrocytes coordinate homeostatic mechanisms in brain. Although the mechanism for this regulation is uncertain, other globally regulated genes, including circadian and clock-controlled transcripts, cycle in different phases in brain regions outside of the suprachiasmatic circadian pacemaker making the cycling of Fabp7 unique. Given the influence that known circadian genes and lipid metabolites exert on complex behavior, and the role that astrocytes play in homeostasis and plasticity, the global cycling of Fabp7 may be intimately related to the maintenance of these processes.

## Materials and Methods

### Subjects and Handling

Mice (C57BL/6) were either purchased directly from Harlan Sprague-Dawley (adult) or taken at specific ages from a breeding colony. A total number of 111 animals were used in this study. Animals were entrained to a 12 hour light, 12 hour dark schedule for at least 5 weeks. For tissue collection, animals were sacrificed by decapitation, their brains dissected, flash frozen at −30°C in 2-methylbutane, and stored at −80°C. For tissue punches of specific regions, brains were embedded in OCT media and sectioned on a Leica (CM3050) cryostat at −25°C as follows (according to Paxinos & Franklin Mouse Brain Atlas, 2^nd^ ed.): 1) TMN: Two sets of two 400 µm bilateral 1 mm diameter punches from Interaural 1.56 to 0.76; 2) LC: Two sets of two 300 µm bilateral 1 mm diameter punches from Interaural −1.40 to −2.00; 3) Pons: Two sets of one 300 µm 2 mm diameter punch from Interaural −1.40 to −2.00. Punches from the same brain region were pooled from each of the animals within the same group, and stored at −80°C until RNA isolation. All animal care and use procedures were in strict accordance with University of Wisconsin IACUC and National Institute of Health guidelines for the humane treatment of laboratory animals.

### Northern Blot Analysis

Northern blotting was performed essentially as described previously [28]. Briefly, brains were dissected and total RNA was isolated using TRIZOL (Invitrogen, Carlsbad, CA), according to the manufacturer's specifications, and stored at −80°C. Prior to loading, each sample was incubated with sample buffer (7.5%Formaldehyde, 43%Formamide, 12% 10XMOPS, 0.12%Ethidium Bromide) for 5 min at 56°C, and then cooled on ice for 5 min. Northern Blots consisted of 1 µg of total RNA per lane and were electrophoresed on a 1.2% agarose gel. RNA was transferred overnight onto a sheet of GeneScreen Plus nitrocellulose (NEN Life Science Products, Boston, MA). DNA templates generated by PCR were labeled with ^32^P using the Megaprime DNA labeling system (Amersham Biosciences, England). Fabp7 forward primer, AGACCCGAGTTCCTCCAGTT, reverse primer, CCTCCACACCGAAGACAAAC. Fabp5 forward primer, CAATTCAGTGAGCGGTCAAA, reverse primer, TAAGCTTGGATCTCCCTTTATTCTATAAAA. Probe was mixed with hybrisol, and incubated overnight at 42°C. Blots were washed three times in 2× SSC (1×SSC = 150 mM NaCl, 15 mM Na citrate, pH, 7.0), 1% SDS at room temperature, then two times for 30 min in 2×SSC, 1% SDS at 50°C, and finally one time for 30 min in 0.5×SSC, 1% SDS at 60°C. Following washes, blots were exposed to a phosphoscreen for 3 days and image analysis was performed using the Storm 860 and ImageQuant 5.2 software (Molecular Dynamics, Sunnyvale, CA). A 983-bp PCR product for glyceraldehyde-3-phosphate dehydrogenase (Gapdh) was generated as described previously [28].

### Quantitative Reverse Transcriptase PCR

All quantitative reverse transcriptase PCR (qRT-PCR) was performed at the University of Wisconsin-Madison Genome Center. Complementary DNA was generated using Superscript II Reverse Transcriptase (SSRII; Invitrogen, Carlsbad, California) as follows: Five µg of total RNA was added to 100 pmole of T7 oligo(dT) primer (GGCCAGTGAATTGTAATACGACTCACTATAGGGAGGCGG-d(T)24) and the reaction volume was brought to 12 µl total volume with PCR grade water. The reaction was incubated at 70°C for 10 minutes in a Mastercycler ep thermocycler (Eppendorf, Hamburg-Eppendorf, Germany) and placed on ice for 2 minutes. Four µl of 5× SSRII buffer, 2 µl of 0.1 M DTT, and 1 µl of 10 mM dNTPs were added and the reaction was incubated in a thermocycler for 2 minutes at 42°C. After incubation, 1 µl of SSRII reverse transcriptase was added and the reaction was incubated at 42°C for 1 hour. cDNA was then quantified using an ND-1000 Spectrophotometer (NanoDrop Technologies, Wilmington, Delaware) and normalized to 20 ng/µl with PCR grade water.

PCR Methods: Individual reactions contained 1× Probes MasterMix (F. Hoffmann-La Roche Ltd, Basel, Switzerland), 0.05 U Uracil-N-Glycosylase, 1× LCGreen Plus (Idaho Technology, Salt Lake City, Utah), 200 nM of each primer and 20 ng of cDNA. Fabp7 mRNA forward primer, CTCTGGGCGTGGGCTTT, reverse primer, TTCCTGACTGATAATCACAGTTGGTT, Fabp7 intron 1 forward primer, CGTACTGTTTTCTTCTTAACTCTGTATTTTG, reverse primer, GATTTCTGAACTATTTTGGAAGTTGCTG. The total reaction volume of 5 µl was overlaid with 10 µ of Chillout Wax (Bio-Rad Laboratories, Hercules, California). All reactions were run on a LightCycler 480 Real-Time PCR System (F. Hoffmann-La Roche Ltd). The cycling parameters were; 50°C for 5 minutes, 95°C for 10 minutes and then 50 cycles of 95°C for 15 seconds and 60°C for 30 seconds. Fluorescence data was collected at a wavelength setting of 450–500 nm. Analysis was performed using both the LightCycler 480's proprietary software and qBASE v1.3.5 (Jan Hellemans, Center for Medical Genetics, Ghent University, Belgium).

### *In Situ* Hybridization

*In situ* hybridization was performed as previously described [71]. Briefly, post-fixed, cryostat sections (20 µm) were pretreated with Proteinase K (0.2 µg/ml; Promega, Madison, WI) and hybridized overnight at 55°C in 150 µl of ^35^S-labeled antisense riboprobe (10,000 cpms/µl). Following posthybridzation washes, sections were exposed to a phosphoscreen for 6 days. Image analysis was performed using the Storm 860 and ImageQuant 5.2 software (Molecular Dynamics Sunnyvale, CA). For densitometric analysis of *in situ* hybridization data, specific regions from a minimum of four sections were averaged per animal per time point, and normalized to background as described previously [72].

Antisense (^35^S- or digoxigenin (DIG) -labeled) riboprobe was made from Fabp7, Nestin or Glial fibrillary acidic protein (GFAP) PCR templates derived from T7-tagged reverse or forward primers, respectively. Fabp7 forward primer: AGACCCGAGTTCCTCCAGTT, reverse primer, CCTCCACACCGAAGACAAAC; Nestin forward primer, GGTCAAGACGCTGGAGGAG, reverse primer, GGAGACCTGGCTCACTTTTTC; GFAP forward primer: AATGCTGGCTTCAAGGAGAC, reverse primer, AGGACAACTGAGCGGACACT. Templates used for *in vitro* transcription were generated using standard PCR conditions. T7 sequence tag extending the reverse primer was GGCCAGTGAATTGTAATACGACTCACTATAGGGAGGCGG. *In vitro* transcription reactions were carried out as described by Promega Biotech (Madison, WI) for ^35^S-labeled riboprobe and by Hoffman-La Roche (Nutley, NJ) for DIG-labeled riboprobe.

For emulsion autoradiography, sections processed for *in situ* hybridization were dipped in NTB3 liquid emulsion (Eastman Kodak, Rochester, New York) under safelight conditions and stored at 4°C for 6 weeks. Processing was as described by the manufacturer. Following development, sections were lightly stained in cresyl violet (0.1%), dehydrated in an ethanol series and mounted. For double *in situ* hybridization analysis, sections were hybridized to both riboprobes, washed as described above and then processed for alkaline phosphatase as described by the manufacturer (Hoffman-La Roche, Nutley, NJ). Sections were then processed with autoradiographic emulsion for 6 weeks.

For analysis of diurnal time points using emulsion autoradiography, all sections within a series were processed under identical conditions in the same run (*N* = 2–5 animals per group). The subgranular zone (SGZ) of hippocampus was analyzed using Image J software (NIH; http://rsb.info.nih.gov). Briefly, the ventral SGZ in dorsal hippocampal sections extending from Interaural 1.62 to 2.22 mm were boxed (minimum of four sections per time point per animal), counts tabulated and background subtracted, and averaged. Final values were calculated by averaging across animals (*N* = 2–5 per group). Prior to emulsion autoradiography, changes in hippocampus were cross-validated using phosphorimaging.

### Western blotting

Samples prepared from whole brain were subjected to SDS-polyacrylamide gel electrophoresis by separating 10 µg protein per lane on a 10–20% gradient pre-cast gel (Biorad, Hercules, CA). Protein was transferred to 0.2 µm Protran nitrocellulose membranes (PerkinElmer, Boston, MA), blocked in 5% dried milk powder in 50 mM Tris-HCl, pH 7.5, 15 mM NaCl, 0.5% Tween-20 (TBST), washed briefly in TBST and incubated in primary antibody in TBST overnight at 4°C. Fabp7 antibody (Chemicon, Temecula, CA) was used at 1∶1000 dilution in TBST, antibody to β-actin (Imgenex, San Diego, CA) was used at 1∶10,000 dilution and anti-glyceraldehyde-3-phosphate dehydrogenase (Gapdh; Imgenex, San Diego, CA), at 1∶5,000 dilution. Blots were then washed 3 times in TBST, incubated for 1 hour in anti-rabbit horse radish peroxidase-conjugated secondary antibody (1∶7500 in TBST; KPL, Gaithersburg, MD), washed 3 times in TBST and incubated in ECL plus Western blotting detection reagent for 5 minutes based on the manufacturers instructions (GE Healthcare, Buckinghamshire, UK). Visualization was performed by chemoluminescence scanning (medium sensitivity, photomultiplier voltage, 600 volts) on a Typhoon 9410 phosphorimager (GE Healthcare, Buckinghamshire, UK). Densitometric analysis was performed using ImageQuant version 5.2.

### Immunohistochemistry

Sections were cut at 50 µm on a vibrotome and stored in 0.01 M phosphate-buffered saline (PBS; pH 7.4) and 0.01% azide solutions until processing. Sections were thoroughly washed in PBS, and endogenase peroxidase activity was quenched by incubation for 10 minutes in PBS containing 10% methanol, 2.5% hydrogen peroxide. Sections were again rinsed, then placed in blocking solution containing 10% normal goat serum (NGS) and 0.2% Triton X-100 for 30 min. Sections were rinsed four times in PBS and then incubated with the primary Fabp7 antibody (1∶2000 dilusion) at 4°C for 72–96 h. After three rinses in PBS, sections were incubated in Alexa Flour goat anti-rabbit 2° antibody (1∶400; Invitrogen, Carlsbad, CA) for 2 h. Following rinsing and coverslipping, photographic images were taken on a Leica DMI 6000 B inverted microscope.

## Supporting Information

Figure S1 Fabp7 messenger (m-) and heteronuclear (hn-) RNA underwent changes in abundance over a 24 hour period. RNA from pons subjected to quantitative RT PCR (qPCR) revealed diurnal changes in Fabp7 mRNA expression that mirrored results from Northern blotting. qPCR for Fabp7 intron 1 (average of TMN, pons, and LC) used as a measure of heteronuclear RNA and therefore transcriptional activity, identified elevated levels of intron 1 near the end of the dark period and after lights-on. Note that increases in Fapb7 hnRNA immediately preceded the apex of Fabp7 mRNA. RNA levels are normalized against Gapdh.
(0.21 MB TIF)

Figure S2 Fabp7 mRNA did not cycle in early postnatal brain. A) RNA collected from postnatal day (P) 6 whole brain analyzed with qPCR identified significantly higher levels of Fabp7 compared with adult whole brain (relative to Gapdh); ***p<0.001 (t-test) B) Analysis by qPCR of Fabp7 mRNA levels across the day revealed statistically significant diurnal changes in adult whole brain that mirrored previous results, while there were no diurnal changes in P6 whole brain (relative to Gapdh, normalized to mean levels within age group); Two-way ANOVA, (p = 0.004); A significant difference in diurnal levels were observed between adult and P6: ZT2.5, **p<0.01; ZT14.5, *p<0.05 (post-hoc Bonferroni). Each value represents the average +/− S.E.M. N = 2–3 per timepoint. Animals were maintained on a 10:14 hour light-dark schedule.
(0.24 MB TIF)
