# Supplementary figures and images for: Brain Fatty Acid Binding Protein (Fabp7) Is Diurnally Regulated in Astrocytes and Hippocampal Granule Cell Precursors in Adult Rodent Brain
Source: PLoS One. 2008 Feb 20;3(2):e1631. doi: 10.1371/journal.pone.0001631 (PMC2238817; doi:10.1371/journal.pone.0001631)

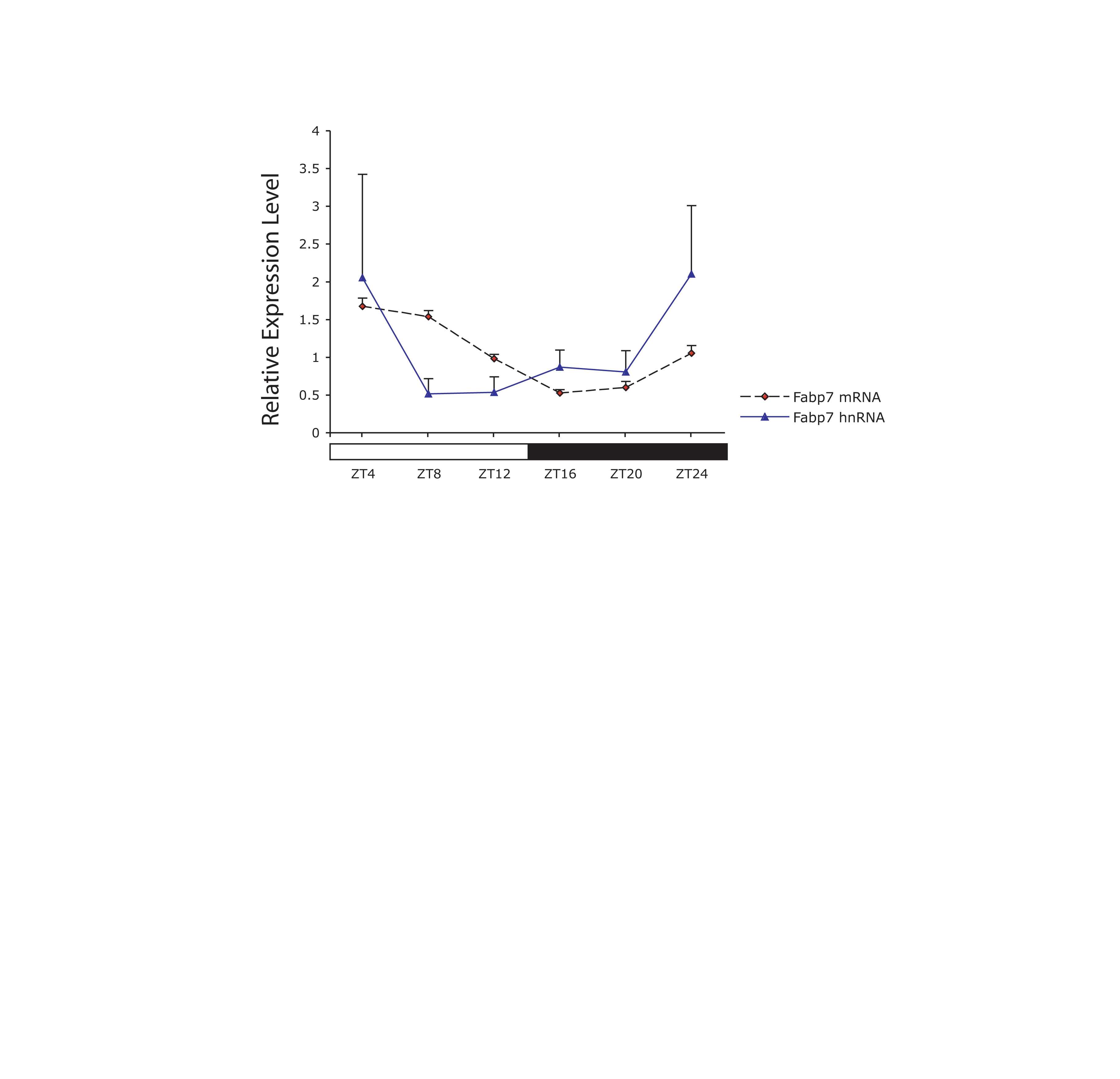

Supplement: Figure S1 — Fabp7 messenger (m-) and heteronuclear (hn-) RNA underwent changes in abundance over a 24 hour period. RNA from pons subjected to quantitative RT PCR (qPCR) revealed diurnal changes in Fabp7 mRNA expression that mirrored results from Northern blotting. qPCR for Fabp7 intron 1 (average of TMN, pons, and LC) used as a measure of heteronuclear RNA and therefore transcriptional activity, identified elevated levels of intron 1 near the end of the dark period and after lights-on. Note that increases in Fapb7 hnRNA immediately preceded the apex of Fabp7 mRNA. RNA levels are normalized against Gapdh. (0.21 MB TIF) [file pone.0001631.s001.tif]

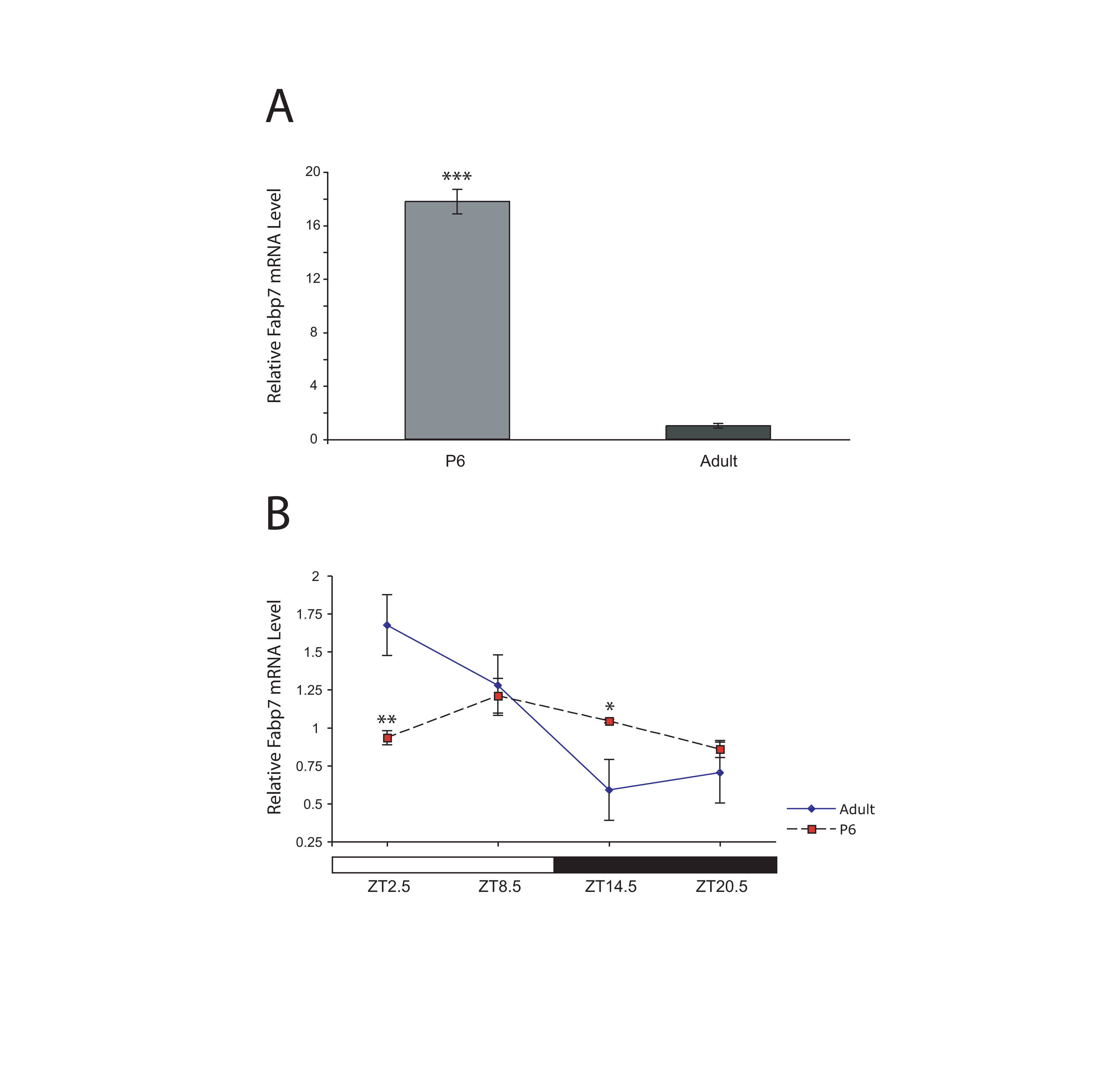

Supplement: Figure S2 — Fabp7 mRNA did not cycle in early postnatal brain. A) RNA collected from postnatal day (P) 6 whole brain analyzed with qPCR identified significantly higher levels of Fabp7 compared with adult whole brain (relative to Gapdh); ***p<0.001 (t-test) B) Analysis by qPCR of Fabp7 mRNA levels across the day revealed statistically significant diurnal changes in adult whole brain that mirrored previous results, while there were no diurnal changes in P6 whole brain (relative to Gapdh, normalized to mean levels within age group); Two-way ANOVA, (p = 0.004); A significant difference in diurnal levels were observed between adult and P6: ZT2.5, **p<0.01; ZT14.5, *p<0.05 (post-hoc Bonferroni). Each value represents the average +/− S.E.M. N = 2–3 per timepoint. Animals were maintained on a 10:14 hour light-dark schedule. (0.24 MB TIF) [file pone.0001631.s002.tif]
